# Case report: Young-onset large vessel ischemic stroke due to hyperhomocysteinemia associated with the C677T polymorphism on *5,10-methylenetetrahydrofolate reductase* and multi-vitamin deficiency

**DOI:** 10.3389/fneur.2023.1183306

**Published:** 2023-05-18

**Authors:** Jiro Fukae, Hiroto Eguchi, Yoichi Wada, Atsuhito Fuse, Rika Chishima, Mitsuyoshi Nakatani, Asuka Nakajima, Nobutaka Hattori, Yasushi Shimo

**Affiliations:** ^1^Department of Neurology, Juntendo University Nerima Hospital, Tokyo, Japan; ^2^Department of Pediatrics, Tohoku University School of Medicine, Sendai, Japan; ^3^Department of Clinical Laboratory, Juntendo University Nerima Hospital, Tokyo, Japan; ^4^Department of Research and Therapeutics for Movement Disorders, Juntendo University School of Medicine, Tokyo, Japan; ^5^Department of Neurology, Juntendo University School of Medicine, Tokyo, Japan

**Keywords:** hyperhomocysteinemia, 5, 10-methylenetetrahydrofolate reductase, cerebral infarction, folic acid, vitamin B12, antiepileptic drug, epilepsy

## Abstract

Hyperhomocysteinemia is an important risk factor for cerebral infarction. Herein, we report on a 30-year-old man previously diagnosed with epilepsy who presented with right hemiplegia and total aphasia. Magnetic resonance imaging showed a fronto-temporal ischemic lesion due to occlusion of the left middle cerebral artery. Clinical testing and imaging demonstrated that he had hyperhomocysteinemia induced by multiple factors including the C677T polymorphism on 5.10-methylenetetrahydrofolate reductase (*MTHFR*), and multiple vitamin deficiencies. The C677T polymorphism on *MTHFR* is closely related to hyperhomocysteinemia and folate deficiency in epileptic patients who are taking multiple anti-convulsants. Given hyperhomocysteinemia can independently cause stroke at a young age, physicians should periodically examine plasma homocysteine and serum folic acid levels in epileptic patients who are on long-term regimens of multiple anti-epileptic drugs.

## Introduction

Epidemiological studies suggest that the incidence of ischemic stroke in young adults (18–50 years old) has increased substantially (1). There are a wide variety of causes for stroke in young adults such as hyperhomocysteinemia, illicit drug use, pregnancy, arterial dissections, patent foramen ovale (PFO), anti-phospholipid syndrome, malignancy, and protein S or C deficiency (1, 2). Moreover, moyamoya disease, that is one of causes of ischemic stroke in young adults, is a specific chronic cerebrovascular occlusive disease first reported in 1957 (3). Hyperhomocysteinemia is an important risk factor for several cardiovascular diseases, including coronary artery disease, peripheral occlusive disease, stroke, and venous thrombosis (4). Increased levels of plasma homocysteine are influenced by both genetic and environmental factors. The C677T polymorphism on the *5.10-methylenetetrahydrofolate reductase (MTHFR)* gene (5, 6), which is identical to c.665C>T in *MTHFR* (NM_005957) gene, and decreased levels of vitamin B6, vitamin B12, and folic acid are associated with elevated homocysteine levels (4). We herein report on a case of young-onset cerebral infarction with hyperhomocysteinemia caused by the C677T polymorphism in the *MTHFR* gene and multiple vitamin deficiencies.

## Case descriptions

At the age of 16, the patient presented with several episodes of generalized tonic-clonic seizure. He was diagnosed with epilepsy based on results of electroencephalography. Despite having started anti-epileptic drugs (AED), seizures occurred once every few months. He was prescribed a regimen of four AEDs such as valproic acid, gabapentin, topiramate, and carbamazepine. At age 28, he often encountered trouble with interpersonal relationships owing to mild developmental delay. At age 30, he expressed abnormal behaviors such as taking large amounts of medications. The day after this incident, he was admitted to our hospital due to disturbed consciousness upon his mother noticing his aberrant behavior. He had no family history of coronary artery disease, stroke, or neuropsychiatric disease. His diet was unbalanced with a predilection for eating meat and avoiding vegetables.

On admission, his blood pressure was 105/64 mmHg, heart rate was 75 beats / min, respiratory rate was 16 breaths / min and body temperature 37.4°C. Physical examination was unremarkable. On neurological examination, his consciousness was somnolence. He had total aphasia and right hemiparesis. The results of laboratory examination were as follows: white blood cell count, 9.6 × 10^9^/L (reference: 3.9–9.7 × 10^9^/L); hemoglobin, 15.2 g/dL (reference: 13.4–17.1 g/dL); platelet count, 315 × 10^9^/L (reference: 153–346 × 10^9^/L); aspartate aminotransferase, 20 IU/L (reference: 5–37 IU/L); alanine aminotransferase, 27 IU/L (reference: 6–43 IU/L); blood urea nitrogen, 8 mg/dL (reference: 9–21 mg/dL); creatine, 0.61 mg/dL (reference: 0.6–1.0 mg/dL); Na, 140 mmol/L (reference: 135–145 mmol/L); K, 3.9 mmol/L (reference: 3.5–5 mmol/L); Cl, 109 mmol/L (reference: 96–107 mmol/L); total cholesterol, 179 mg/dL (reference: 150–219 mg/dL); high-density lipoprotein (HDL) -cholesterol, 30 mg/dL (reference: 40–70 mg/dL); HbA1c, 4.9% (reference: 4.6–6.2%). Protein C and S were within normal limits. International normalized ratio (INR) was 1.28 (reference: 0.85–1.15), and d-dimer was 0.5 μg/mL (reference: 0–1). His thyroid function was within normal limits. Immunological examination for autoimmune disorders, including for anti-nuclear antibody, anti-ribonucleoprotein (RNP), anti-SSA, anti-SSB, proteinase (PR) 3-anti-neutrophil cytoplasmic antibody (ANCA), myeloperoxidase (MPO)-ANCA antibodies, and anti-cardiolipin antibodies were all negative. Plasma homocysteine level was markedly increased to 74.1 nmol/ml (reference: 3.7–13.5 nmol/ml), and methionine level was 28.1 nmol/ml (reference: 18.9–40.5 nmol/ml). Folate, vitamin B12, and pyridoxal were all below-normal ranges (folate: 1.0 ng/ml [reference: > 4 ng/ml], vitamin B12 135 pg/ml [reference: 180–914 pg/ml], pyridoxal 3.2 ng/ml [reference: 6–40 ng/ml]).

**Figure 1:**
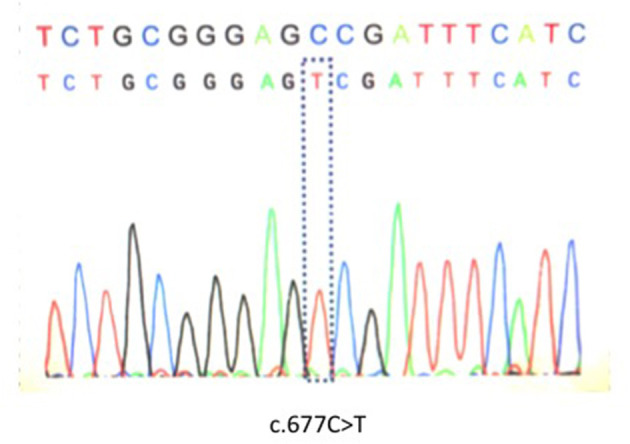
Genetic analysis identified homozygous C677T polymorphism in the *MTHFR* gene.

**Figure 2:**
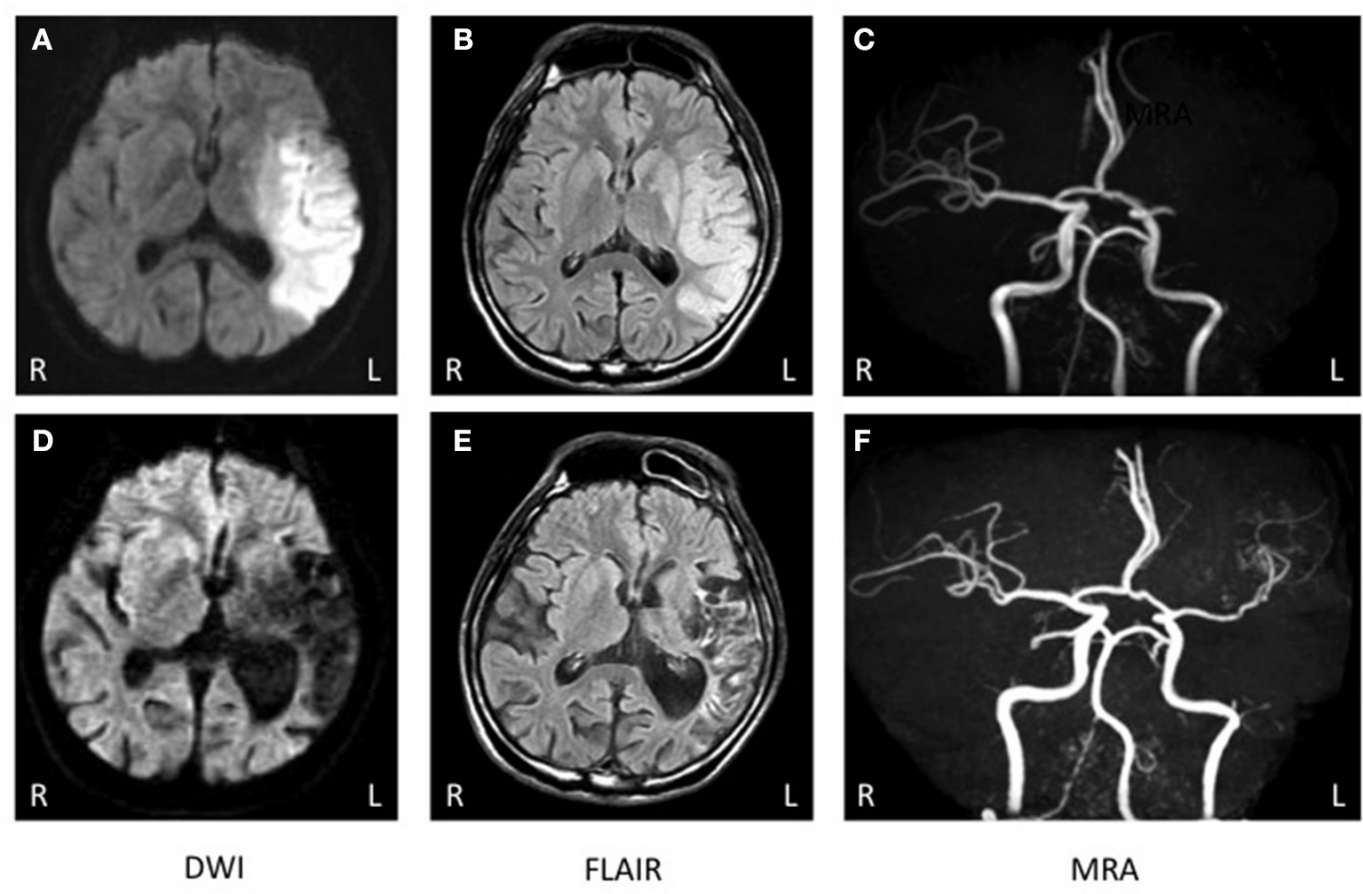
**(A–C)** Brain magnetic resonance imaging (MRI) at onset. **(A, B)** Brain MRI of diffusion-weighted imaging (DWI) and FLAIR exhibited a high intensity area in the left fronto-temporal region. **(C)** Brain magnetic resonance angiography (MRA) showed occlusion of the left middle cerebral artery (MCA). **(D–F)** MRI 2 years after onset. **(D, E)** Brain MRI of diffusion-weighted imaging (DWI) and FLAIR exhibited a low intensity area in the left fronto-temporal region. **(F)** Brain MRA showed that the left MCA was recanalized but there were no findings of double lumen or string and pearls signs.

Genetic analysis was performed after written informed consent was obtained as MTHFR enzyme deficiency was suspected. Genetic analysis identified C677T polymorphism that appeared to be homozygous (Figure 1). Brain computed tomography showed a low intensity area in the region of the left middle cerebral artery (MCA) (data not shown). On diffusion weighted imaging and FLAIR of brain magnetic resonance imaging, a large high intensity area was seen in the left MCA territory (Figures 2A, B). Additionally, magnetic resonance angiography showed that the MCA was disrupted in the M1 segment (Figure 2C). Holter electrocardiogram revealed no arrhythmia or atrial fibrillation. Transesophageal echocardiography showed no thrombus in the left atrium and no right-left shunt. He was diagnosed with atherothrombotic cerebral infarction. Administration of aspirin 200 mg/day that was started upon diagnosis was decreased to 100 mg/day after 3 weeks. To treat his hyperhomocysteinemia, folic acid (15 mg/day), vitamin B1 (150 mg/day), vitamin B6 (150 mg/day), and vitamin B12 (150 mg/day) were administered. On this course of treatment, serum folic acid, vitamin B1, and vitamin B6 increased, and serum homocysteine decreased to 5.8 nmol/ml. His consciousness gradually improved to being alert and his total aphasia ameliorated to motor aphasia. Three months after admission, his condition improved to a point at which he was able to carry out activities of daily living. Therefore, he was discharged from our hospital.

After discharge, he continued a regimen of AEDs such as valproic acid, gabapentin, topiramate, and levetiracetam instead of carbamazepine. During the 3 years after discharge, he was without seizure, and electroencephalography confirmed that he had no epileptic activity. Administration of folic acid, vitamin B1, vitamin B6, and vitamin B12 was continued for hyperhomocysteinemia. His plasma homocysteine level was maintained within normal limits (5.8–10.5 nmol/ml). Two years after discharge, a brain MRI showed no new infarction (Figures 2D, E). MR angiography revealed that the occluded MCA had been recanalized, but no double lumen sign or string and pearls sign were detected (Figure 2F). For the infarct, he was treated with clopidogrel and had no recurrence of ischemia.

## Discussion

We herein reported on a patient with epilepsy and developmental delay during adolescence who suffered an ischemic stroke at age 30. He was devoid of common risk factors of atherosclerosis such as hypertension, diabetes mellitus, and hyperlipidemia. Based on the results of laboratory examination, neuroimaging, and physiological function tests, his stroke was not caused by cardiogenic embolism, paradoxical embolism or vascular malformation. Hyperhomocysteinemia was the only factor associated with the large vessel occlusion.

Meta-analyses have revealed a consistent association between plasma homocysteine levels and atherosclerotic disorders (7, 8). Additionally, several studies have demonstrated that high plasma homocysteine levels are associated with small vessel stroke (9–11). In contrast, Tantirittisak et al. (12) reported that abnormal homocysteine levels were more pronounced in a group with large vessel stroke compared to small vessel stroke. Jeong et al. (13) showed that an increased level of plasma homocysteine was associated with internal carotid artery occlusion in patients with ischemic stroke. Taken together, these reports suggest that elevated plasma homocysteine is associated with not only small vessel but also large vessel stroke. As our patient had only hyperhomocysteinemia as a vascular risk factor, we conclude that his ischemic event was caused by a hyperhomocysteinemia independently progressed atherosclerosis leading to arterial occlusion. The exact mechanism by which increased levels of homocysteine lead to the development of atherosclerosis is still unknown. Clinical and experimental findings have shown that hyperhomocysteinemia can increase oxidative stress and change the homeostasis of the endothelium (14). At later stages of the atherosclerotic process, homocysteine increases platelet activation and aggregation and causes coagulation abnormalities, thereby promoting vascular occlusion (15). Furthermore, hyperhomocysteinemia may induce abnormal proliferation of smooth muscle cells and increase inflammatory processes that induce the development of atherosclerosis and trigger thrombosis (4).

**Figure 3:**
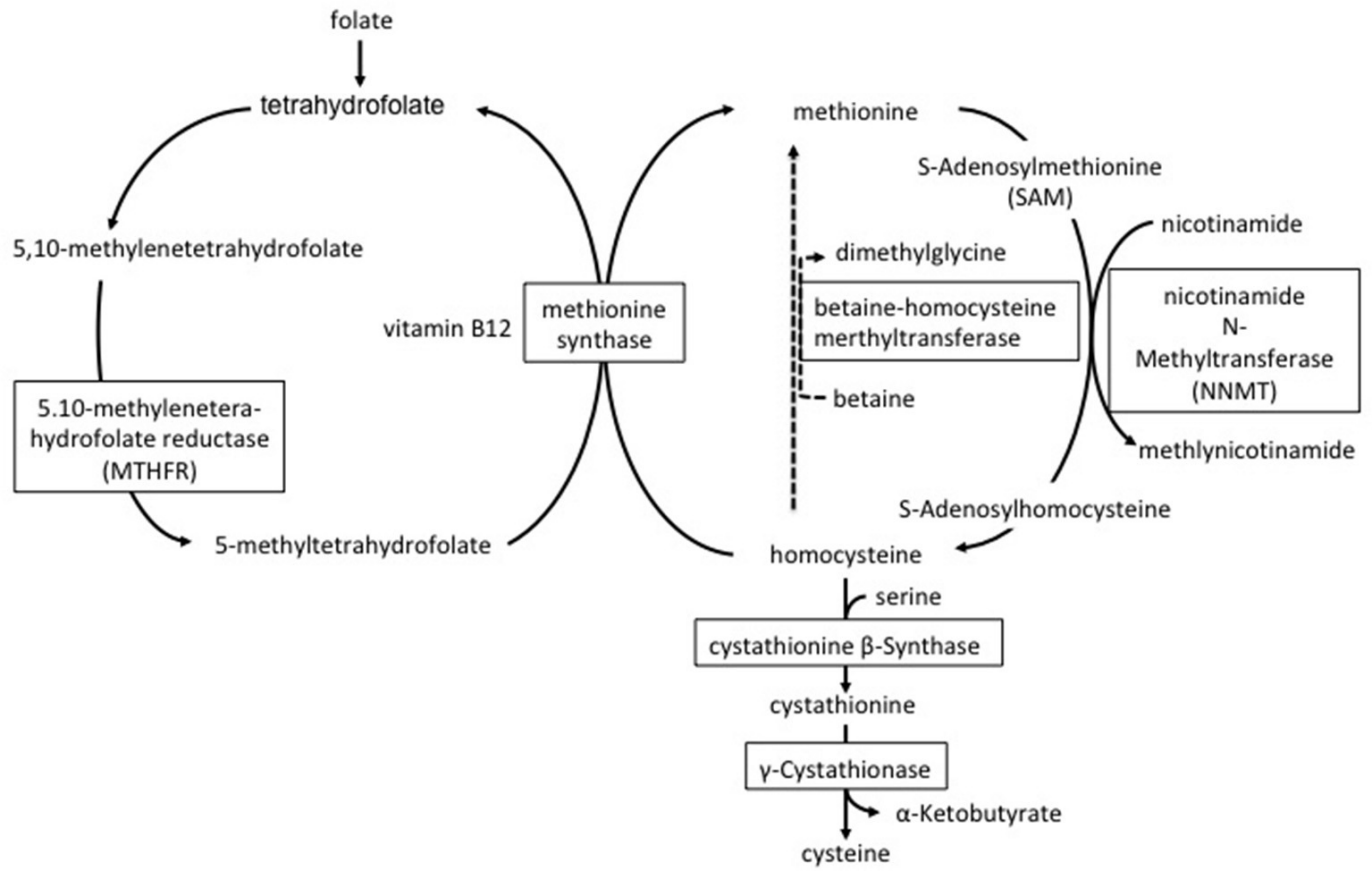
Metabolism of homocysteine.

Plasma homocysteine levels are influenced by genetic and environmental factors. Mutations in multiple genes are known to contribute to *cystathionine beta-synthase, MTHFR* and *nicotinamide N-methyltransferase (NNMT)* (5, 6, 16, 17). Cystathionine beta-synthase deficiency impairs the conversion of homocysteine to cystathionine and leads to both homocysteine and methionine accumulation (16) (Figure 3). MTHFR converts 5,10-methylenetetrahydrofolate to 5-methyltetrahydrofolate that produces methyl donor groups for the conversion of homocysteine to methionine (Figure 3). Impaired activity of the MTHFR enzyme leads to increased plasma homocysteine but not to increased plasma methionine. Our patient showed high serum homocysteine and normal methionine levels; therefore, we hypothesized that his MTHFR activity was decreased and found the C677T polymorphism in his *MTHFR* gene. As the C677T polymorphism of the *MTHFR* gene reduces the thermostability of the MTHFR enzyme, MTHFR enzyme activity under this homozygous polymorphism is 50–60% lower at 37°C compared with normal non-mutated controls (6, 18). The prevalence of the C677T polymorphism in the *MTHFR* gene is variable depending on ethnicity and nationality. For example, the percentage of the Japanese population with the homozygous mutation was reported at approximately 11% (19, 20). The A/G polymorphism NNMT (rs694539) is also known to be associated with hyperhomocysteinemia (17). NNMT is an enzyme involved in the synthesis of S-Adenosylhomocysteine (SAH); it catabolizes nicotinamide and other pyridine compounds in a reaction that uses the methyl group generated during the conversion of S-Adenosylmethionine to SAH (Figure 3) (21). In a Japanese study, there were no differences in plasma homocysteine concentration between the NNMT AA+AG and GG genotypes, suggesting that this polymorphism is not a major determinant of plasma homocysteine concentration in the Japanese men (22). However, only when together with the NNMT GG genotype and other confounding factors, such as age, folate deficiency, and/or MTHFR C677T, were they associated with an elevation of plasma homocysteine (22). In our case, unfortunately, we did not perform a NNMT gene analysis. It is possible that the patient had the NNMT GG genotype because his plasma homocysteine was elevated significantly.

As for environmental factors, sex, smoking, and low vitamin levels are associated with levels of serum homocysteine. Meta-analyses have revealed that carbamazepine, valproate sodium, and phenytoin are associated with an increase in plasma levels of homocysteine (23–25). Additionally, studies have demonstrated that these medications significantly decrease serum levels of folic acid vitamin B6 and vitamin B12 (26, 27). These vitamins play important roles in the metabolism of homocysteine (Figure 3). In our patient, the use of carbamazepine and valproate sodium may have decreased serum folate and vitamin B6 and consequently caused hyperhomocysteinemia. As a result, we changed the patient from taking carbamazepine to levetiracetam which has no effect on the levels of plasma homocysteine.

Hyperhomocysteinemia is an important risk factor for stroke due to atherosclerosis, and venous thrombosis (4). In the treatment of ischemic stroke due to atherosclerosis in patients with hyperhomocysteinemia, lowering of the homocysteine concentration through administration of folic acid and vitamin B12 is the most important therapy. Several previous studies have suggested that a lowering of homocysteinemia therapy prevented stroke recurrence (28, 29). Another previous report demonstrated that patients with hyperhomocysteinemia avoided death and altered their medical progress through the use of antihypertensive therapy and the administration of aspirin (30). These results may suggest that a combination of anti-platelet drugs and reducing homocysteine therapy is effective in preventing the progression of atherosclerosis caused by hyperhomocysteinemia. Hyperhomocysteinemia is also an important risk factor for thrombosis, both intracranially and in the venous return of the lower limbs (31, 32). These findings suggest that hyperhomocysteinemia increases the risk of ischemic stroke due to cerebral venous thrombosis and paradoxical embolism. A previous report shows that lowering homocysteine concentration was effective for preventing recurrent venous thrombosis (33). In the treatment of strokes due to venous thrombosis, anti-coagulant therapy is effective (34). From these viewpoints, a combination of anti-coagulant therapy and a reduction of homocysteine therapy was effective in the prevention of strokes due to venous thrombosis caused by hyperhomocysteinemia.

In conclusion, the C677T mutation is closely related to hyperhomocysteinemia and folate deficiency in epileptic patients taking multiple anti-convulsants (27). Therefore, physicians should periodically examine plasma homocysteine and folic acid levels in epileptic patients who are on a long-term regimen of multiple AEDs. In the case of well-controlled patients, a supplement of folic acid could be prescribed to normalize plasma homocysteine levels. If patients' seizures are uncontrolled, physicians should consider using other AEDs such as lamotrigine or levetiracetam which do not affect plasma levels of homocysteine (35, 36).

## Data availability statement

The datasets presented in this article are not readily available because of ethical and privacy restrictions. Requests to access the datasets should be directed to the corresponding author.

## Ethics statement

Written informed consent was obtained from the individual(s), and minor(s)' legal guardian/next of kin, for the publication of any potentially identifiable images or data included in this article.

## Author contributions

JF performed the data research and wrote the manuscript. JF and HE treated the patient. YW performed gene analysis. AF, RC, MN, and AN supported the clinical interpretation. NH and YS was critically involved in the theoretical discussion and composition of the manuscript. All authors read and approved the final version of the manuscript.
